# Advances in dry eye disease: from immunopathological mechanisms to emerging ophthalmic drug delivery systems

**DOI:** 10.3389/fmed.2026.1780733

**Published:** 2026-04-01

**Authors:** Ning Yang, Jiancheng Mu, Feng Xu, Wanyue Guo, Chuhuan Sun, Bosen Peng, Junhan Xiang, Lixing Deng, Wei Fan

**Affiliations:** 1 Department of Ophthalmology, West China Hospital of Sichuan University, Chengdu, Sichuan, China; 2 Chengdu AIDI Eye Hospital, Chengdu, Sichuan, China

**Keywords:** dry eye disease, immunopathology, nanomedicine, ocular surface inflammation, ophthalmic drug delivery, precision therapy

## Abstract

Dry eye disease (DED) is a multifactorial disorder of the lacrimal functional unit and ocular surface that leads to ocular discomfort, visual disturbance, and tear film instability. It affects a substantial proportion of the global population and is driven by a complex interplay of immune dysregulation, environmental stressors, and systemic factors. Accumulating evidence indicates that immune-mediated inflammation is central to DED pathogenesis and is closely intertwined with oxidative stress, autophagy imbalance, pyroptosis, apoptosis, ferroptosis, viral infection, and alterations in the ocular surface microbiota, ultimately disrupting ocular surface homeostasis. Despite the availability of multiple therapeutic options, current treatments often fail to achieve sustained symptom relief, largely due to short ocular residence time, limited bioavailability, and insufficient targeting of underlying inflammatory mechanisms. In recent years, innovative ophthalmic drug delivery systems–including nanoparticles, hydrogels, liposomes, microspheres, and emerging gene-based platforms–have been developed to enhance drug retention, improve ocular bioavailability, and enable controlled and targeted therapy. This review provides an updated and integrative overview of the immunopathological mechanisms underlying DED and critically summarizes recent advances in ophthalmic drug delivery technologies. By linking disease mechanisms with translational delivery strategies, we highlight how emerging delivery systems may overcome the limitations of conventional therapies and facilitate precision, long-acting, and patient-centered treatment for DED. These insights may inform future therapeutic development and guide the clinical translation of innovative treatment strategies for this increasingly prevalent ocular surface disease.

## 1 Introduction

Dry eye disease (DED), also known as keratoconjunctivitis sicca, is a multifactorial ocular surface disorder characterized by tear film instability and ocular discomfort (1). It is primarily caused by lacrimal gland dysfunction, insufficient tear secretion, or excessive evaporation and is classified into aqueous-deficient, evaporative, or mixed subtypes (2). Histopathological changes include corneal and conjunctival epithelial damage, goblet cell loss, and meibomian gland dysfunction, ultimately leading to disruption of tear film homeostasis.

According to the TFOS DEWS II Definition and Classification Report, the diagnosis of DED requires the presence of patient-reported symptoms together with objective evidence of tear film homeostasis disturbance. Clinical parameters such as reduced fluorescein breakup time (FBUT) or decreased Schirmer I test values are commonly used to reflect tear film instability or aqueous deficiency; however, specific diagnostic thresholds (e.g., FBUT ≤ 5 s or Schirmer I ≤ 5 mm/5 min) may vary across guideline contexts and should not be interpreted as standalone diagnostic criteria (3–5).

The lacrimal functional unit (LFU), composed of the ocular surface, lacrimal glands, and their neural connections, maintains tear film homeostasis (6). Structural or functional impairment of any component may disrupt this balance and initiate inflammatory cascades. Multiple risk factors contribute to DED development, including aging, female sex, genetic predisposition, systemic diseases, hormonal imbalance, psychological stress, and environmental exposures such as air pollution and low humidity (7–10). Drug-induced DED has also been increasingly recognized (11, 12).

Reported global prevalence varies widely, ranging from approximately 5% to over 50%, largely reflecting heterogeneity in diagnostic criteria, study design, population characteristics, and geographic region (13). Meta-analyses provide more conservative pooled estimates, with a 2022 analysis reporting an overall prevalence of approximately 8%–10% worldwide (8). Higher rates are frequently observed in Asian populations and in older individuals.

Dry eye disease imposes a substantial socioeconomic burden: in China, the annual direct cost per patient is approximately USD 465, and in the United States, the total healthcare burden is estimated at USD 3.8 billion annually (14, 15). In recent years, lifestyle changes and increased digital device use have contributed to rising prevalence among younger populations (16–18).

Despite the availability of multiple therapeutic modalities, such as artificial tears, anti-inflammatory drugs, and punctal occlusion, many patients experience persistent symptoms and unsatisfactory outcomes. This is largely due to short ocular residence time, low drug bioavailability, and the inability to effectively address underlying inflammation. These challenges highlight the need for new treatment approaches.

This review aims to provide an updated overview of the pathophysiological mechanisms of DED and to highlight recent advances in innovative ophthalmic drug delivery systems. By integrating current mechanistic insights and technological innovations, this article seeks to support the development of more effective and patient-centered therapeutic strategies.

## 2 Pathophysiological mechanisms of dry eye disease

Dry eye disease (DED) presents with diverse clinical manifestations; however, these symptoms arise from a shared underlying pathophysiological cascade. According to the TFOS DEWS II framework, DED is fundamentally characterized by loss of tear film homeostasis and hyperosmolar stress, which initiate epithelial injury and immune-mediated inflammatory amplification. Inflammation plays a central role in perpetuating ocular surface damage and is closely interconnected with oxidative stress, autophagy dysregulation, and various forms of programmed cell death, including pyroptosis, apoptosis, and ferroptosis. In addition, viral infection and alterations of the ocular surface microbiome have been increasingly recognized as potential modulators of immune responses and tear film stability, although their precise causal contributions remain under investigation.

### 2.1 Loss of tear film homeostasis: a DED-specific pathophysiological cascade

Within this DED-specific pathophysiological framework, tear film instability leads to increased osmolar stress on the ocular surface epithelium, triggering intracellular stress signaling pathways such as MAPK and NF-κB activation. This epithelial stress response promotes the production of pro-inflammatory cytokines and chemokines, which in turn amplify local immune activation.

Hyperosmolarity-induced epithelial damage disrupts barrier integrity and enhances exposure of danger-associated molecular patterns (DAMPs), facilitating recruitment of innate immune cells and subsequent activation of adaptive immune responses. Over time, persistent inflammatory signaling contributes to goblet cell loss, meibomian gland dysfunction, and tear film lipid layer abnormalities, further exacerbating tear instability.

In parallel, neurosensory abnormalities emerge due to chronic inflammation and epithelial injury, altering corneal nerve sensitivity and contributing to symptom–sign discordance observed in many DED patients. These interconnected processes establish a self-perpetuating vicious cycle in which tear film instability, hyperosmolar stress, immune activation, and epithelial damage continuously reinforce one another.

Within this DED-specific causal framework, mechanisms such as oxidative stress, autophagy dysregulation, pyroptosis, apoptosis, ferroptosis, and microbiota imbalance can be understood not as isolated pathways, but as modulators that intensify epithelial injury and inflammatory amplification.

**FIGURE 1:**
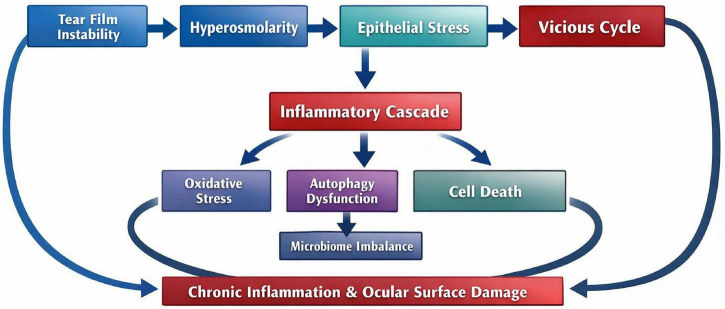
Pathophysiological mechanism of DED. This schematic outlines the DED-specific pathophysiological cascade. Tear film instability leads to hyperosmolarity and epithelial stress, which drive both an inflammatory cascade and a vicious cycle that feeds back to tear film instability. The inflammatory cascade promotes oxidative stress, autophagy dysfunction, and cell death (e.g., pyroptosis, apoptosis, ferroptosis); autophagy dysfunction also contributes to microbiome imbalance. These processes converge to sustain chronic inflammation and ocular surface damage, which in turn aggravate tear film instability and complete the self-perpetuating loop.

Within this DED-specific causal framework, mechanisms such as oxidative stress, autophagy dysregulation, pyroptosis, apoptosis, ferroptosis, and microbiota imbalance can be understood not as isolated pathways, but as modulators that intensify epithelial injury and inflammatory amplification. This cascade, from tear film instability and hyperosmolarity through epithelial stress, inflammatory amplification, and the vicious cycle, to the downstream roles of oxidative stress, autophagy dysfunction, programmed cell death, and microbiome imbalance in sustaining chronic inflammation and ocular surface damage, is summarized in Figure 1.

### 2.2 Immune-mediated inflammatory amplification in DED

Disruption of ocular immune homeostasis damages the ocular surface barrier and promotes chronic inflammation, eventually leading to DED (18). Inflammation and DED reinforce each other to form a vicious cycle of epithelial injury and tear film instability. Both innate and adaptive immune responses contribute to this process. Immune cells such as neutrophils, macrophages, NK cells, and dendritic cells (DC1, DC2), as well as CD4^+^ T helper cells (Th1/Th17), are deeply involved (19). Inflammatory mediators, including TNF-α, IL-1β, IL-6, and matrix metalloproteinases (MMPs), are up-regulated during DED progression (20). A summary of the key molecular mechanisms, signaling pathways, and potential therapeutic targets involved in immune-mediated inflammation and other DED-related processes is presented in Table 1.

**TABLE 1:** Molecular mechanisms and therapeutic targets in dry eye disease (DED).

Mechanism	Key molecular pathways/biomarkers	Pathophysiological role in DED	Representative experimental/clinical evidence	Potential therapeutic targets or agents
Immune-mediated inflammation	TNF-α, IL-1β, IL-6, IL-17, IFN-γ, MMP-9, CXCL8, TGF-β, MUC5AC (18–20, 29).	Central immune activation involving Th1/Th17 imbalance, cytokine overproduction, epithelial barrier disruption	Overexpression of cytokines and matrix metalloproteinases in ocular surface tissues and tears (19, 20).	Anti-inflammatory cytokine blockers, MMP inhibitors, cyclosporine, tacrolimus
Innate immunity	Neutrophils, macrophages (M1), NK cells, DC1/DC2, complement C3a/C5a (23–28).	Early ocular immune activation and complement-driven tissue injury	Increased neutrophil infiltration and complement activation in DED models (24, 28).	Complement inhibitors, IFN-γ antagonists, NK cell modulators
Adaptive immunity	CD4^+^ T cells, Th1/Th2/Th17/Treg, MHC-II, ICOSL, VISTA, PD-1H, GITRL (28, 30–38).	Antigen-specific response sustaining chronic inflammation	High CD4^+^ T-cell infiltration; cyclosporine efficacy supports adaptive immune role (28, 31).	Calcineurin inhibitors, IL-17 antagonists, T-cell costimulatory blockers
Oxidative stress	ROS, NF-κB, NLRP3, SOD1, 4-HNE, MDA, COX-2, HO-1 (39–42).	ROS accumulation triggers NF-κB activation and inflammation	Elevated oxidative markers and reduced antioxidant enzymes in DED patients (40–42).	Antioxidants (SkQ1, C-NAC, CoQ10, Vitamin B12, sodium hyaluronate + fluorometholone) (43–46).
Autophagy dysregulation	LC3, p62, mTOR, AMPK, TFEB, ROS (47–51).	Impaired autophagy reduces epithelial repair and increases inflammation	Upregulated LC3 and autophagy flux modulation affects corneal injury and repair (47–49).	Rapamycin, trehalose, LYN-1604, 5-HT1A antagonists
Pyroptosis	NLRP3, caspase-1/4/5/11, GSDMD, IL-1β, IL-18 (52–57).	Inflammasome activation induces lytic cell death and cytokine release	ROS–NLRP3–IL-1β axis promotes epithelial damage; GSDMD deletion alleviates disease (54–56).	MCC950 (NLRP3 inhibitor), GSDMD blockers, IL-1β/IL-18 inhibitors
Apoptosis	Caspase-3/8, p53, Bcl-2, ERK1/p38 MAPK (23, 26, 58, 59).	Epithelial and goblet cell loss, chronic inflammation maintenance	Enhanced caspase and MAPK signaling in DED tissues; Qishen Granule reduces apoptosis (23, 26, 59).	MAPK inhibitors, anti-apoptotic herbal compounds (Qishen Granule), Bcl-2 modulators
Ferroptosis	Iron accumulation, GPX4, NRF2–AKR1C1, lipid peroxidation markers (60, 61).	Iron-dependent oxidative injury and lipid peroxidation impair tear secretion	Inhibition of ferroptosis restores tear secretion and reduces oxidative injury (60, 61).	Ferrostatin-1, liproxstatin-1, NRF2 activators, VIP supplementation
Viral infection	NF-κB, TLR3/TRIF, RIG-I/RIP-1, IL-6, CXCL1, CXCL6 (62–69).	Viral nucleic acids activate innate defense and trigger chronic ocular inflammation	Upregulated NF-κB and cytokines in SARS-CoV-2, HTLV-1, and EBV infections (64–68).	Antiviral and immunomodulatory therapy (corticosteroids, TLR3 inhibitors, NF-κB blockers)

APC, antigen-presenting cell; DC, dendritic cell; GSDMD, gasdermin D; HO-1, heme oxygenase 1; LC3, microtubule-associated protein 1A/1B-light chain 3; MMP, matrix metalloproteinase; NF-κB, nuclear factor kappa-light-chain-enhancer of activated B cells; NRF2, nuclear factor erythroid 2–related factor 2; ROS, reactive oxygen species; TFEB, transcription factor EB; VIP, vasoactive intestinal peptide.

Beyond intrinsic inflammatory cascades, immune dysregulation may also arise from systemic immune modulation. Immune checkpoint inhibitors (ICIs), increasingly used in oncology to enhance antitumor immunity, have been associated with immune-related adverse events involving the ocular surface, including dry eye–like symptoms and inflammatory keratoconjunctivitis. A retrospective analysis of 962 ICI-treated patients reported ophthalmic immune-related adverse events in 1.1% of patients during the first year of treatment, with manifestations including conjunctivitis, ophthalmoplegia, and orbital inflammation (21). Uveitis represents the most common ocular adverse event associated with ICIs, particularly in melanoma patients, while dry eye disease occurs in approximately 1%–4% of patients receiving ICI therapy (22). These clinical observations illustrate how disruption of systemic immune tolerance can precipitate ocular surface immune imbalance. Recognition of iatrogenic immune activation reinforces the importance of precisely understanding the immune pathways described in DED and highlights the need for balanced immunomodulatory strategies.

#### 2.2.1 Innate immune response

The innate immune response serves as the first line of defense against external stimuli such as desiccation, pathogens, or mechanical injury. It plays a crucial role in maintaining ocular surface integrity by preventing microbial invasion and toxin penetration through the epithelial barrier (23). In DED, innate immune cells–including neutrophils, natural killer (NK) cells, and monocytes/macrophages–are key mediators of early inflammation. Neutrophil infiltration is markedly increased on the ocular surface of patients with severe aqueous-deficient DED but is rarely observed in normal conjunctival tissue (24). Activated NK cells secrete interferon-γ (IFN-γ), which aggravates conjunctival epithelial injury, induces goblet cell loss, and promotes Th1 cell differentiation (25, 26). Monocytes preferentially polarize toward the pro-inflammatory M1 phenotype, leading to the sustained production of cytokines that amplify local inflammation and tissue damage (27).

Dendritic cells (DC1/DC2) interact with IFN and TNF to amplify receptor expression and sustain the inflammatory state. Serum transfer from DED mice to nude mice induces similar pathology through complement activation (C3a/C5a, C3b/C5b), inflammatory-cell infiltration, and cytokine production (28). In addition, proteins such as CXCL8, MUC5AC, S100, MMP-3, macrophage inflammatory protein-2, epidermal growth factor, lactoferrin, and TGF-β are altered in DED ocular surfaces (29). These findings confirm that dysregulated innate immunity triggers ocular surface inflammation and tissue damage.

#### 2.2.2 Adaptive immune response

Adaptive immunity involves antigen-specific T-cell activation in regional lymph nodes and subsequent migration to the ocular surface. The high abundance of CD4^+^ T cells in DED and the efficacy of cyclosporine therapy both indicate the central role of adaptive immunity. Proliferation and differentiation of T cells into Th1, Th2, Th17, and Treg subsets amplify pathological immune loops (30, 31). In diabetes-associated DED, advanced glycation end products (AGEs) promote dendritic-cell maturation and enhance CD4^+^ T-cell activation, stimulating excessive cytokine release.

The ocular surface expresses high levels of MHC-II, IL-6, and IL-17, facilitating antigen presentation and T-cell recruitment to the conjunctiva and cornea (28). Activated T cells further stimulate antigen-presenting cells (APCs), releasing IL-1β, TNF-α, and IL-6, which disrupt epithelial integrity (31–33). Several surface molecules such as ICOSL, B7-H2, B7RP-1 (34), VISTA, PD-1H (35), GITRL (36), and galectin-9 (37) modulate antigen-specific T-cell responses. Thymic damage before allogeneic hematopoietic stem-cell transplantation reduces central immune tolerance, demonstrating that thymus-mediated regulation is essential for ocular surface immune balance (38).

#### 2.2.3 Mechanistic basis of immune-mediated inflammation

The immunopathogenesis of DED can be divided into four phases: initiation, amplification, recruitment, and tissue damage. Inflammation interacts with several molecular pathways, including oxidative stress, autophagy, pyroptosis, apoptosis, ferroptosis, and viral infection.

(a) Oxidative Stress

Oxidative stress promotes secondary immune activation and worsens ocular surface injury. In SOD1-knockout mice, oxidative damage and inflammatory infiltration in lacrimal glands increase markedly, while tear secretion decreases, confirming its value as a DED model (39). Various animal models such as Mev-1^–^/^–^, Nrf2^–^/^–^, M3R^–^/^–^, and others induced by environmental or surgical factors demonstrate that oxidative stress is a core pathological driver (40). Clinical studies show that elevated tear oxidative stress correlates with increased inflammatory mediators and polymorphonuclear leukocyte infiltration (41). Lipid peroxidation markers such as 4-HNE and malondialdehyde (MDA) are elevated in patients with non-Sjögren DED and correlate with tear breakup time, Schirmer score, corneal staining, and goblet cell density (42).

Reported oxidative-stress biomarkers include oxidant markers (4-HNE, 8-OHdG, COX-2, ROS, MDA) and antioxidant markers (SOD, CAT, GSH, HO-1, vitamins), providing useful indicators for disease progression (40). Antioxidant therapies show promising results: the mitochondrial-targeted antioxidant SkQ1 protects corneal tissue (43); chitosan-N-acetylcysteine (C-NAC) improves OSDI scores and reduces redness (44); combined sodium hyaluronate 0.1% + fluorometholone 0.1% and hyaluronic acid 0.15% + vitamin B12 eye drops enhance anti-inflammatory and antioxidant effects (45, 46).

(b) Autophagy

Autophagy regulates survival and activity of inflammatory cells such as lymphocytes, neutrophils, and macrophages, and its imbalance contributes to ocular inflammation. In Sjögren’s-syndrome DED, conjunctival epithelial autophagy is dysregulated. In murine DED models, modulating autophagy can reduce lacrimal gland dysfunction and epithelial injury by suppressing inflammation. Ma et al. demonstrated that autophagy inducer LYN-1604 enhanced LC3 expression, cell migration, and corneal repair, whereas inhibitor 3-MA had opposite effects (47). Similar findings in xerophthalmia rats confirmed that autophagy induction promotes epithelial survival and wound healing. *In vitro*, rapamycin-induced autophagy protects human corneal epithelial cells (HCECs) under hyperosmotic stress (48).

In benzalkonium-chloride-induced DED mice, activation of 5-HT1A receptors increased ROS production and impaired autophagy, while 5-HT1A antagonists reduced ROS and inflammation (49). Trehalose, an autophagy enhancer, suppressed Akt-TFEB signaling and produced anti-inflammatory effects independent of NF-κB activation (50, 51). Altogether, regulating autophagy may serve as a novel therapeutic approach for DED.

(c) Pyroptosis

Pyroptosis is an inflammatory form of programmed cell death characterized by cell swelling, membrane rupture, and cytokine release (52). It is mediated by caspase-1 through the classical inflammasome pathway or by caspase-4/5/11 upon LPS binding (53). In DED patients, NLRP3 inflammasome components and caspase-1 activity are elevated on the ocular surface (54). Reactive oxygen species activate NLRP3, leading to caspase-1 auto-activation and IL-1β maturation in DED models (55). The ROS–NLRP3–IL-1β axis thus contributes to ocular inflammation (54). Wei et al. reported that deletion of gasdermin D (GSDMD), the substrate cleaved by active caspases, alleviates desiccating-stress-induced epithelial injury; NLRP12 cooperates with NLRC4 to trigger GSDMD-dependent pyroptosis in corneal mucosa (56). Shao et al. found that LPS-activated caspase-4 cleaves pro-IL-18, releasing mature IL-18 and promoting inflammation via the caspase-4/5–IL-18 axis (57).

(d) Apoptosis

Apoptosis is markedly enhanced in DED, leading to cell loss in lacrimal acini, conjunctival and corneal epithelium, and corneal endothelium, while lymphocyte apoptosis is suppressed, prolonging inflammation (58). The apoptotic cascade involves caspase-8, IFN-γ, p53, and Bcl-2-family proteins (23, 26). Zhao et al. demonstrated that Qishen Granule down-regulates ERK1/p38 MAPK phosphorylation and reduces IL-1β, IL-8, and caspase-3 levels in HCECs, effectively limiting inflammation and apoptosis (59).

(e) Ferroptosis

Ferroptosis is an iron-dependent form of cell death driven by lipid peroxidation and oxidative stress. In a unilateral corneal-nerve-cut model, ferroptosis and decreased vasoactive intestinal peptide (VIP) expression were observed in lacrimal glands, whereas VIP or ferrostatin-1 supplementation restored tear secretion (60). In corneal epithelial cells, iron accumulation and lipid peroxidation are enhanced under hyperosmolarity, while inhibition of ferroptosis reduces oxidative injury. NRF2 activation promotes AKR1C1 expression to limit cell damage and inflammation (61). These findings indicate that ferroptosis contributes to DED pathogenesis and may represent a therapeutic target.

(f) Viral Infection

Patients with Sjögren-related DED frequently show viral infections such as Epstein–Barr virus (EBV), hepatitis C virus (HCV), human immunodeficiency virus (HIV), and human T-cell lymphotropic virus (HTLV-1) (62, 63). When infected by these viruses or by SARS-CoV-2 and HBV, the host immune system recognizes viral nucleic acids and activates innate defense pathways, inducing inflammation and structural damage on the ocular surface (64, 65). In corneas, limbus, and sclera infected with SARS-CoV-2, NF-κB activation up-regulates RELB, IL-6, CXCL1, and CXCL6 (66). HTLV-1 infection activates CD4^+^ T cells to secrete pro-inflammatory cytokines, causing ocular inflammation that can be improved by topical or oral corticosteroids (67). EBV infection increases NF-κB subunit activation and TLR3 expression, while NF-κB signaling in HCECs depends on TLR3/TRIF and RIG-I/RIP-1 pathways (68). HCV infection elevates IL-6, IL-8, NO, and TNF-α levels, stimulating corneal epithelial and conjunctival fibroblast activity (69). Clinically, viral-associated immune dysregulation is most relevant in specific DED subgroups, such as patients with Sjögren’s syndrome or systemic autoimmune conditions. While viral persistence does not account for the majority of common DED cases, these findings suggest that immune activation in selected patients may be influenced by viral triggers. In routine clinical practice, antiviral therapy is not typically indicated; however, recognition of virus-related immune modulation may help guide individualized immunoregulatory strategies in complex cases.

In summary, the pathogenesis of DED involves multiple overlapping mechanisms including immune activation, oxidative stress, autophagy imbalance, and various forms of programmed cell death. These interconnected pathways disrupt ocular surface homeostasis and create a persistent inflammatory loop, providing multiple potential targets for therapeutic intervention.

### 2.3 The ocular surface microbiota

In addition to immune-mediated inflammation, the ocular surface microbiota, an integral part of the local microenvironment, plays a crucial role in the onset and progression of dry eye disease (DED). The ocular surface harbors a stable community of commensal bacteria that maintains immune tolerance and epithelial integrity. Disturbance of this microbiota, known as dysbiosis, can initiate or aggravate ocular surface inflammation.

A metagenomic analysis by Li et al. (70) revealed distinct microbial compositional differences between healthy individuals and DED patients. In the general population, the relative abundance of dominant phyla–Proteobacteria (51.7%), Firmicutes (16.9%), Bacteroidetes (13.6%), Actinobacteria (6.1%), and Cyanobacteria (1.7%)–was altered in DED patients, where Proteobacteria decreased to 47.6% and Bacteroidetes increased to 16.5%, suggesting a microbial shift associated with disease severity (70). In patients with Meibomian Gland Dysfunction (MGD), a major evaporative subtype of DED, both bacterial isolation rate and species diversity were significantly higher than in healthy controls (71). Dong et al. further identified *Staphylococcus* and *Sphingomonas* as characteristic taxa of MGD. The degree of meibomian gland atrophy positively correlated with the abundance of *Staphylococcus*, indicating that this genus may contribute to gland obstruction and lipid layer instability (72).

Mechanistically, bacterial dysbiosis contributes to DED through several pathways: (1) disruption of the antibacterial epithelial barrier, (2) breakdown of symbiotic equilibrium between ocular microbiota and host defense, (3) altered vitamin synthesis and mucin production, and (4) activation of Toll-like receptors (TLRs) and pro-inflammatory cytokine cascades (73). The resulting immune activation perpetuates local inflammation and tear film instability. For patients with mild microbial imbalance, the use of artificial tears and lubricant formulations may help restore ocular surface homeostasis and support recovery of commensal flora.

In summary, increasing evidence underscores the significance of the ocular microbiome in DED pathophysiology. However, direct human evidence remains limited, and most findings are derived from small-scale or cross-sectional studies. Future research should focus on longitudinal and mechanistic investigations linking microbial alterations to specific immune and epithelial responses, which may ultimately inform microbiota-targeted therapies for DED management (70–73).

## 3 Innovative drug delivery systems

**FIGURE 2:**
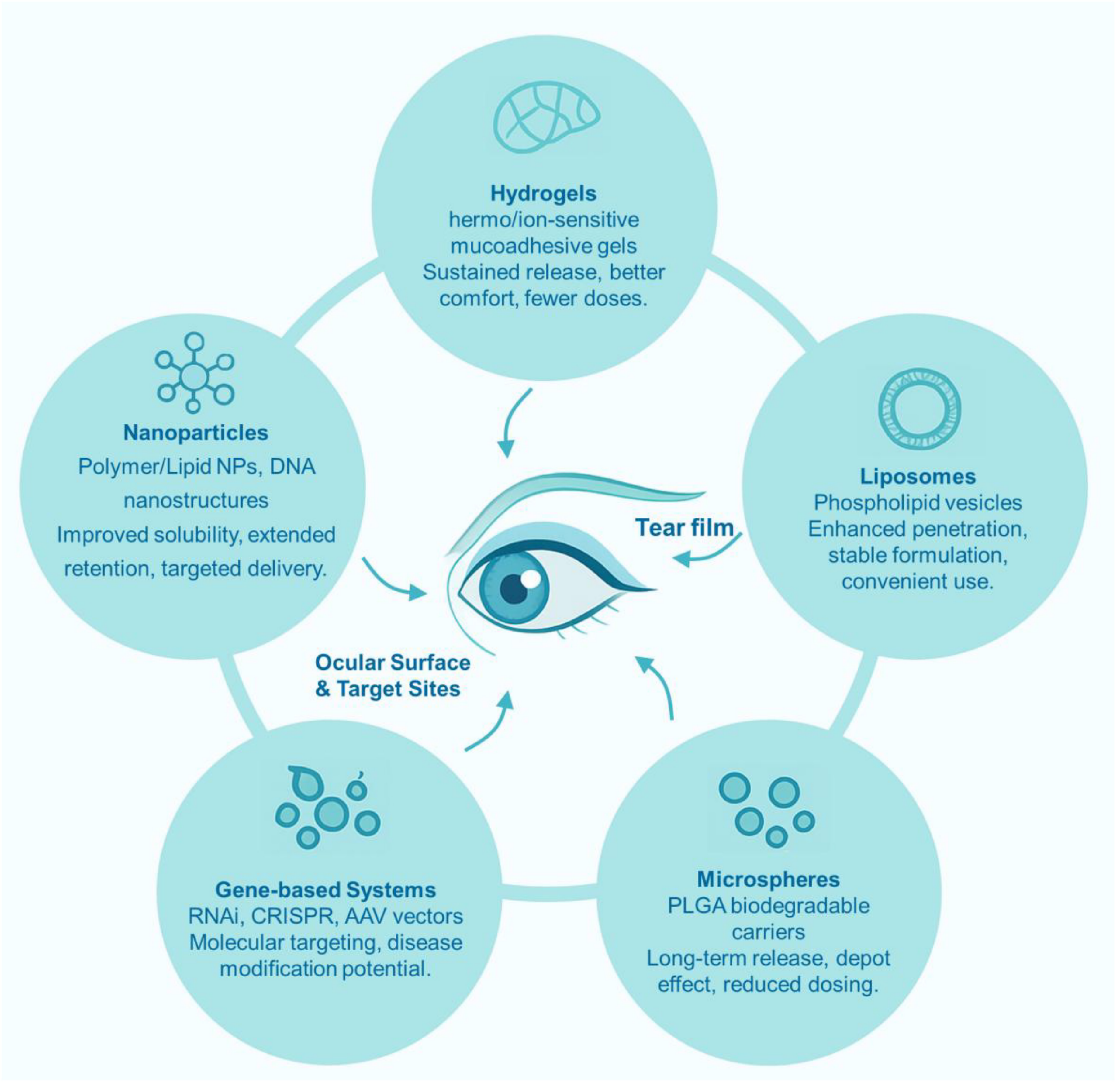
Overview of emerging ophthalmic drug delivery systems for dry eye disease (DED). This schematic illustrates five key platforms designed to enhance ocular surface drug delivery. Hydrogels are thermo- or ion-sensitive mucoadhesive gels that provide sustained drug release, improved comfort, and reduced dosing frequency. Liposomes, composed of phospholipid vesicles, facilitate enhanced corneal penetration through the tear film and offer stable and convenient formulations. Microspheres (e.g., PLGA biodegradable carriers) enable long-term drug release and depot effects, reducing the need for frequent administration. Nanoparticles (including polymer-, lipid-, and DNA-based nanostructures) improve drug solubility, extend ocular retention, and allow targeted delivery to the ocular surface. Finally, gene-based systems (such as RNAi, CRISPR, and AAV vectors) offer molecular-level targeting and disease-modifying potential, representing a promising direction for next-generation therapies in DED management.

Understanding the pathophysiological mechanisms of DED is crucial for the development of effective therapeutic strategies. As discussed above, the multifactorial nature of DED requires drug delivery systems capable of addressing multiple pathological pathways simultaneously. Conventional treatments include artificial tears, anti-inflammatory drugs, cyclosporine, corticosteroids, mucin secretagogues, vitamin A, autologous serum, tacrolimus eye drops, Thermaeye Plus, and oral traditional Chinese medicine (33, 74–76). Among them, topical eye drops account for nearly 90% of the formulations currently in use. However, physiological barriers, poor corneal permeability, and low drug solubility limit the bioavailability of most ophthalmic drugs. Therefore, the development of innovative delivery systems such as nanoparticles, hydrogels, liposomes, and microspheres has become a major research focus (77). To better illustrate the diversity and mechanisms of these emerging ophthalmic delivery technologies, Figure 2 summarizes five major categories of emerging ophthalmic drug delivery systems for dry eye disease (DED). These include hydrogels, which are thermo- or ion-sensitive mucoadhesive gels that enable sustained and controlled drug release, improve patient comfort, and reduce dosing frequency; liposomes, which are phospholipid vesicles enhancing corneal penetration through the tear film and providing stable, convenient formulations; microspheres, such as PLGA-based biodegradable carriers, which achieve long-term release and depot effects with reduced dosing needs; nanoparticles, including polymer-, lipid-, and DNA-based nanostructures, which enhance solubility, extend ocular retention, and facilitate targeted delivery; and gene-based systems, utilizing RNAi, CRISPR, or AAV vectors to achieve molecular-level targeting and disease modification. Together, these innovative systems aim to improve ocular surface bioavailability, extend drug residence time, and deliver sustained therapeutic efficacy in DED management.

### 3.1 Nanoparticles for immunomodulatory and anti-inflammatory therapy in DED

In dry eye disease (DED), nanoparticle-based delivery systems are primarily intended to enhance ocular surface retention of anti-inflammatory agents and enable sustained modulation of immune pathways. Because DED is characterized by tear film instability and rapid tear clearance, conventional eye drops often exhibit limited corneal bioavailability. Nanoparticle platforms may partially overcome these limitations by prolonging residence time and facilitating controlled drug release (78, 79).

Nanocarrier systems originally developed for anterior-segment infections or inflammatory conditions–such as PLGA-based and lipid-based nanoparticles–have demonstrated improved stability, corneal penetration, and sustained pharmacokinetic profiles in experimental ocular models (80, 81). Although these studies were not conducted specifically in DED populations, they provide proof-of-concept evidence that controlled-release nanostructures can enhance ocular surface exposure.

For DED-specific translation, nanoparticle design should prioritize surface retention, mucoadhesion, compatibility with tear film dynamics, and minimal disruption of the lipid layer. Sustained-release systems may be particularly suitable for DED-relevant payloads such as cyclosporine A or other immunomodulators (79). Overall, nanoparticle platforms represent a promising but still translationally evolving strategy for DED-targeted therapy, and further disease-specific optimization and clinical validation are required (78).

### 3.2 Hydrogel systems for sustained lubrication and immune regulation in DED (condensed version)

Hydrogel-based ophthalmic delivery systems are particularly relevant to DED because they simultaneously address tear film instability and chronic ocular surface inflammation. By forming in situ gels upon ocular contact, hydrogels can prolong drug residence time, enhance lubrication, and reduce dosing frequency, thereby improving therapeutic adherence (82, 83).

Hydrogel platforms initially developed for antimicrobial or regenerative ophthalmic applications provide proof-of-concept evidence for sustained precorneal retention and controlled drug release (84, 85). Although many of these systems were not specifically designed for DED, their ability to mitigate rapid tear clearance is directly applicable to ocular surface inflammatory disorders.

For DED-specific translation, formulation strategies should prioritize mucoadhesion, tear osmolarity compatibility, optical transparency, and minimal visual disturbance. In addition to functioning as passive drug carriers, certain thermosensitive or ion-sensitive hydrogels may facilitate localized immunomodulator delivery while supporting epithelial repair processes (86).

Compared with nanoparticle systems, hydrogels offer superior surface retention and lubrication effects but may face limitations in diffusion-controlled release and long-term structural stability. Nevertheless, their combined mechanical and biological properties make them promising candidates for DED-oriented drug delivery, pending further disease-specific optimization and clinical validation (87).

### 3.3 Liposomal formulations in inflammatory and evaporative DED (refined version)

Liposomes are particularly relevant in DED management due to their phospholipid bilayer structure, which resembles components of the natural tear film lipid layer. This structural similarity enhances ocular surface compatibility and may contribute to stabilization of the tear film, especially in evaporative DED characterized by lipid layer deficiency (88, 89).

Several liposomal systems initially developed for antimicrobial or posterior-segment applications provide proof-of-concept evidence for enhanced corneal penetration and prolonged precorneal retention (90, 91). Although these studies were not conducted specifically in DED populations, they demonstrate the capacity of liposomal carriers to improve pharmacokinetics and facilitate sustained delivery of hydrophilic or hydrophobic agents at the ocular surface.

Clinically, liposome-containing spray formulations have been applied in mild-to-moderate evaporative DED, underscoring the translational feasibility of lipid-based ocular surface supplementation (89). However, beyond tear film stabilization, future development should focus on integrating liposomal carriers with targeted immunomodulatory or antioxidant payloads to more directly address inflammatory amplification pathways in DED.

Compared with hydrogel systems, liposomes may provide superior integration with the tear film lipid layer but can face formulation stability and storage challenges. Their future therapeutic value in DED likely lies in combining lipid-layer restoration with controlled delivery of disease-modifying agents, although further clinical validation is required (88).

### 3.4 Microsphere-based depot systems for sustained ocular surface therapy in DED (refined version)

Microsphere-based delivery systems offer a depot-style therapeutic strategy that may be particularly advantageous in moderate-to-severe DED requiring prolonged immunomodulation. Given the chronic and relapsing nature of DED, sustained-release platforms that reduce dosing frequency could improve adherence and minimize fluctuations in ocular surface inflammation.

Many microsphere formulations, particularly PLGA-based systems, were originally developed for corticosteroid delivery or posterior-segment inflammatory conditions (92–94). These studies provide proof-of-concept evidence of controlled release kinetics, biodegradability, and favorable safety profiles. However, for DED-specific translation, microsphere systems would require adaptation toward surface-targeted rather than intraocular delivery, optimization of particle size to minimize visual disturbance, and compatibility with tear film dynamics.

Microspheres may be especially suitable for sustained delivery of immunomodulatory agents such as cyclosporine A or tacrolimus, where prolonged exposure could stabilize inflammatory activity while reducing peak-dose irritation (79, 95). Compared with hydrogels and nanoparticles, microspheres can offer more robust long-term release but may face challenges related to formulation viscosity and patient comfort. Future DED-oriented microsphere platforms should therefore aim to balance sustained immunoregulation with ocular surface tolerability, and further disease-specific validation is needed (78).

### 3.5 Gene-based and emerging precision delivery strategies for DED (condensed version)

Gene-based and precision delivery approaches represent an emerging research direction in DED, particularly for cases characterized by persistent immune dysregulation. Because DED involves complex interactions among inflammatory, oxidative, and cell death pathways, upstream gene modulation has been proposed as a potential strategy to complement conventional pharmacologic therapies.

RNA interference, CRISPR/Cas systems, and viral-vector platforms have demonstrated targeted gene regulation in retinal and corneal disorders (96, 97). Although these technologies provide proof-of-concept feasibility, their application to DED remains largely experimental and would require careful adaptation to ocular surface tissues, with emphasis on transient modulation and safety in inflamed environments.

Potential strategies may include modulation of pro-inflammatory cytokines, antioxidant pathways, or inflammasome-related signaling. However, concerns regarding delivery specificity, immunogenicity, and long-term safety persist. At present, gene-based interventions in DED should be regarded as exploratory, and further preclinical validation is necessary before clinical translation can be considered.

### 3.6 Translational considerations: aligning drug delivery platforms with the pathophysiological priorities of DED (condensed version)

Effective translation of advanced drug delivery systems into DED therapy requires alignment with the disease’s core pathophysiological priorities. Unlike posterior-segment disorders, DED is fundamentally characterized by ocular surface inflammation, tear film instability, epithelial barrier disruption, and neurosensory abnormalities.

Accordingly, DED-oriented delivery platforms should emphasize: (1) prolonged ocular surface residence rather than deep intraocular penetration; (2) compatibility with tear film osmolarity and lipid layer integrity; (3) sustained modulation of immune and oxidative stress pathways; and (4) minimal visual disturbance with high patient tolerability.

Different delivery systems may preferentially match specific therapeutic objectives. Nanoparticles can enhance epithelial penetration and targeted immunomodulation; hydrogels integrate lubrication with sustained release; liposomes support lipid-layer stabilization; microspheres enable depot-style long-term modulation; and gene-based approaches remain exploratory options for upstream precision targeting.

Linking platform selection to DED subtypes–such as aqueous-deficient, evaporative, or immune-predominant variants–may facilitate a more rational and precision-guided therapeutic strategy in future development.

### 3.7 Comparative evaluation of drug delivery platforms in DED and translational challenges

Although multiple advanced delivery platforms have been explored for ocular applications, their translational suitability in DED depends on alignment with disease-specific therapeutic priorities. Because DED primarily involves ocular surface instability and chronic inflammatory amplification, platforms emphasizing surface retention, tear film compatibility, and sustained yet tolerable immunomodulation are more likely to achieve meaningful clinical impact.

Each delivery strategy offers distinct strengths and limitations. Nanoparticles facilitate targeted epithelial penetration but face challenges regarding long-term biocompatibility and potential accumulation in ocular tissues; hydrogels combine lubrication with controlled release yet may suffer from inconsistent gelation kinetics and visual disturbance; liposomes support lipid-layer stabilization but exhibit limited shelf stability and batch-to-batch variability; microspheres enable depot-style modulation but require optimization to avoid foreign body sensation; and gene-based approaches remain exploratory options for upstream intervention with unresolved safety concerns regarding immunogenicity and off-target effects. However, no single platform fully addresses the multifactorial pathophysiology of DED.

Beyond technical performance, several translational barriers impede clinical adoption of these advanced delivery systems. First, regulatory pathways for combination products (device-drug or biologic-delivery system) remain complex and variable across jurisdictions, potentially delaying market entry. Second, manufacturing scalability and batch reproducibility pose significant hurdles, particularly for nanoparticles and liposomes where size distribution and surface characteristics critically influence efficacy and safety. Third, the economic burden of novel delivery platforms may limit accessibility; without compelling cost-effectiveness data, healthcare systems may be reluctant to adopt advanced formulations over conventional, albeit less effective, therapies. Fourth, the paucity of head-to-head clinical trials comparing different delivery platforms within the same DED subtype prevents evidence-based platform selection. Finally, patient acceptability factors–including comfort, visual clarity, and dosing convenience–require rigorous evaluation in real-world settings rather than controlled laboratory conditions.

Future development may therefore favor subtype-specific or combinational strategies that integrate mechanical tear film stabilization with precise immune modulation, coupled with systematic approaches to address these translational barriers through regulatory science advances, standardized manufacturing protocols, and appropriately designed comparative effectiveness studies.

## 4 Conclusions and future perspectives

Inflammation plays a central role in the initiation and progression of dry eye disease (DED), and anti-inflammatory therapies remain a cornerstone of current management. However, most existing treatments rely on frequent topical administration or invasive interventions, which are often associated with poor patient adherence, ocular irritation, infection risk, and limited drug bioavailability. These limitations underscore the need for non-invasive, sustained, and targeted therapeutic strategies that more effectively address the underlying immunopathology of DED.

Recent advances in ophthalmic drug delivery systems–including nanoparticles, hydrogels, liposomes, and microspheres–have demonstrated significant potential to enhance ocular surface drug retention, improve bioavailability, and reduce dosing frequency. By enabling controlled and site-specific drug release, these platforms offer promising solutions to overcome the pharmacokinetic barriers of conventional eye drops.

Recent work has further expanded this field through the development of injectable and in situ-forming biomaterials designed to provide sustained lubrication while simultaneously modulating local immune responses. For example, Zhang et al. developed a composite hydrogel combining DNase I-loaded chitosan nanoparticles with silk fibroin that modulates neutrophil extracellular traps and reduces pathological neovascularization by approximately 70% after corneal chemical injury (98). Wang et al. demonstrated that a subconjunctival cyclosporine A/Lifitegrast sustained-release membrane using poly(lactate-co-ε-caprolactone) can release both drugs for at least 1 month, significantly improving conjunctival hyperemia and corneal staining in a rabbit dry eye model (99). Furthermore, intrinsic immunomodulatory hydrogels have been engineered to actively engage with the immune system and optimize tissue repair in chronic inflammatory conditions (100–112). Unlike conventional artificial tears that primarily offer transient symptomatic relief, these next-generation biomaterials form stable, long-lasting interfaces on the ocular surface and incorporate anti-inflammatory or immune-regulatory functionalities. Such systems represent an important conceptual shift toward disease-modifying strategies that address both tear film instability and underlying immunopathology in DED. Nevertheless, challenges such as formulation stability, drug-loading efficiency, long-term safety, and clinical scalability remain to be addressed before widespread clinical adoption. Looking ahead, DED research is increasingly moving toward a precision-medicine paradigm that integrates immunology, biomaterials science, and data-driven approaches. Emerging multi-omics technologies and artificial intelligence–based analytical tools provide new opportunities to identify disease subtypes, predictive biomarkers, and individualized therapeutic targets. In parallel, growing evidence highlights the role of the ocular surface microbiome in modulating immune responses and tear film stability, suggesting that microbiota-targeted interventions may complement existing pharmacological strategies.

Future progress in DED management will depend on multidisciplinary collaboration and well-designed multicenter clinical studies to validate novel drug delivery platforms and establish standardized evaluation frameworks. For example, sustained-release cyclosporine formulations delivered via hydrogel or nanoparticle systems may improve adherence in moderate-to-severe inflammatory DED. Liposomal platforms designed to stabilize the tear film lipid layer may be particularly beneficial in evaporative DED, while depot-based microsphere systems could offer long-acting immunoregulation in refractory cases. These concrete applications illustrate how mechanistic understanding can translate into subtype-specific therapeutic strategies. By bridging immunopathological insights with innovative delivery technologies and precision-guided strategies, the field is poised to move beyond symptomatic relief toward mechanism-based, long-acting, and personalized therapies that can meaningfully improve patient outcomes and quality of life.
